# Uncovering psychologically mediated pathways to cardiovascular diseases: depressive symptoms as a mediator between childhood maltreatment and single or multiple cardiovascular disease comorbidities

**DOI:** 10.3389/fpsyt.2025.1560961

**Published:** 2025-10-09

**Authors:** Peilin Yu, Hong Zhang, Xinxin Zhang, Ping Zeng, Chu Zheng, Ke Wang

**Affiliations:** ^1^ Department of Biostatistics, School of Public Health, Xuzhou Medical University, Xuzhou, Jiangsu, China; ^2^ Department of Emergency, Children’s Hospital Affiliated to Xuzhou Medical University, Xuzhou, Jiangsu, China; ^3^ Center for Medical Statistics and Data Analysis, Xuzhou Medical University, Xuzhou, Jiangsu, China; ^4^ Jiangsu Engineering Research Center of Biological Data Mining and Healthcare Transformation, Xuzhou Medical University, Xuzhou, Jiangsu, China

**Keywords:** depression symptoms, childhood maltreatment, cardiovascular diseases, mediation analysis, comorbidity

## Abstract

**Objective:**

Childhood maltreatment (CM) increases the risk of cardiovascular disease (CVD), but the mediating mechanism of depressive symptoms in this process has not been fully elucidated. To further elucidate the potential mechanisms of depression in the association between CM and CVD, this study aimed to investigate the mediating role of depressive symptoms in a UK biobank cohort.

**Methods:**

Correlation scales for CM types and depressive symptoms were first collected. Additionally, the diagnostic types of CVD were identified. Meanwhile, after controlling for child socioeconomic and demographic factors measured at baseline, we developed logistic regression models to analyze correlations and marginal effects among the three. Next, we used mediated causality modeling in all cohorts to assess whether depressive symptoms explained the association between CM and CVD. Finally, we further explored its indirect effects in multiple CVD comorbidities and gender groups.

**Results:**

A total of 114,707 participants were included in the analysis, of which 50.14% reported CM. Our study demonstrated a strong association between CM scores and increased risk of CVD or depressive symptoms. Mediation analysis indicated that depressive symptoms accounted for 31.03% to 55.28% of the total effect for single CVD comorbidities, and 22.93% to 36.46% for multiple CVD comorbidities. Interestingly, across gender groups, males had a higher proportion of depressive symptoms mediating the association between the two.

**Conclusion:**

The research results remind us to pay attention to the impact of psychological factors on the CM population, so as to reduce the incidence rate of different types of CVDs.

## 1 Introduction

Although tertiary prevention measures have significantly reduced the incidence of cardiovascular disease (CVD), the number of deaths due to CVD has steadily climbed over the decades to 18.6 million deaths and the number of diagnosed cases to 523 million (1). Previous studies have shown that early negative life factors contribute to an increased risk of having CVD, such as high cholesterol levels and high blood pressure in children (2). Recently, a large body of evidence now suggests that childhood maltreatment (CM), which is predominantly based on family characteristics, is associated with chronic adverse outcomes of the organism, especially different types of CVD (3), mental illness (4), diabetes (5), etc.

CM, an important component of adverse childhood experiences (ACEs), is defined as a variety of injuries suffered by children before the age of 18 years as a result of acts or omissions of parents or other caregivers (6). Interestingly, there is even a dose-response relationship between increased CM and CVDs such as myocardial infarction (7). Nowadays, due to the high rate of diagnosis of CM and the widespread damage to cardiovascular health and life expectancy in adults (8), more and more studies have begun to focus on the individual mechanisms of influence between CM and CVD in European and American countries (9). The American Heart Association (AHA) reported a scientific statement of a hypothesized pathway for a potential relationship between the two, suggesting that psychological factors may indirectly contribute to the association (10).

Adverse emotions induced by CM can impair psychological development, leading to psychological (11)and emotional dysregulation (12).This activates the HPA axis, which chronically elevates cortisol and increases the risk of depression and anxiety (13). It also affects neurohormones, coagulation and platelet function, ultimately promoting CVD (14). Previous studies have reported on the link between the above three (15), for example, depression was found to play an indirect role in different types of ACE and CVD in a recent study using the Adverse Childhood Experience-International Questionnaire (ACE-IQ) in middle-aged and older Chinese adults (16). Nevertheless, several reports have yielded mixed results in this regard due to differences in the assessment of variables and selection of populations (17). For instance, no mediating role for depression was observed in another study of U.S. adolescents/adults (17). Chronic atherosclerosis driven by hypertension often co-occurs with other CVDs, making multimorbidity common. Because different comorbidity patterns may reflect different mechanisms, we examined the association between CM and CVD comorbidity. The well-known high prevalence of depression in females and the variability of the association between CM and CVD by gender (18) make it increasingly necessary to investigate in depth whether depression plays a role as a mediating variable in the progression of CM to CVD in different sex groups, as well as the size of the mediating ratio. While prior studies have examined links between adverse childhood experiences and cardiovascular disease, there remains a gap in longitudinal evidence particularly from UK cohorts on the mediating role of depression and the moderating effects of gender and comorbidity patterns in these pathways. Nevertheless, few studies have explored the extent to which the mediating variable depression mediates the effect of CM scores (CM scores [0–5] were assessed using the 5-item CTS, covering five domains, with scores based on Likert thresholds and showing good validity and reliability) with single or multiple CVD comorbidities and different gender groups.

Therefore, the present study, utilizing longitudinal follow-up data from the UK Biobank, was dedicated to exploring the possible causal effects of CM scores on different types of CVD, and to further explore the potential mediating role of depressive symptoms in both. Given that different types of CVD often co-occur (19), we ultimately also included these comorbidities as study endpoints to compare the relative importance of depressive symptoms in mediation analyses. Meanwhile, we conducted an exploratory analysis of the differences in the proportion of depression-mediated mediators across gender, to more fully understand the role of gender in the observed associations. In conclusion, our research findings may findings support a hypothesized pathway in which depressive symptoms mediate part of the CM–CVD association.

## 2 Methods

### 2.1 Data and participants

This study utilized data from the UK Biobank, which recruited 502,401 participants between 2006-2010. The database is an ongoing prospective population-based cohort study designed to accurately and comprehensively assess the environmental, psychosocial, genetic, and nongenetic factors associated with exposure and outcome and to further analyze the complex associations between these factors (20). Subsequently, online questionnaires on psychological issues (21) were added, including the Childhood Trauma Screener (CTS) (for CM) and the Patient Health Questionnaire 9-item (PHQ-9) (for depressive symptoms) (22).

**Figure 1:**
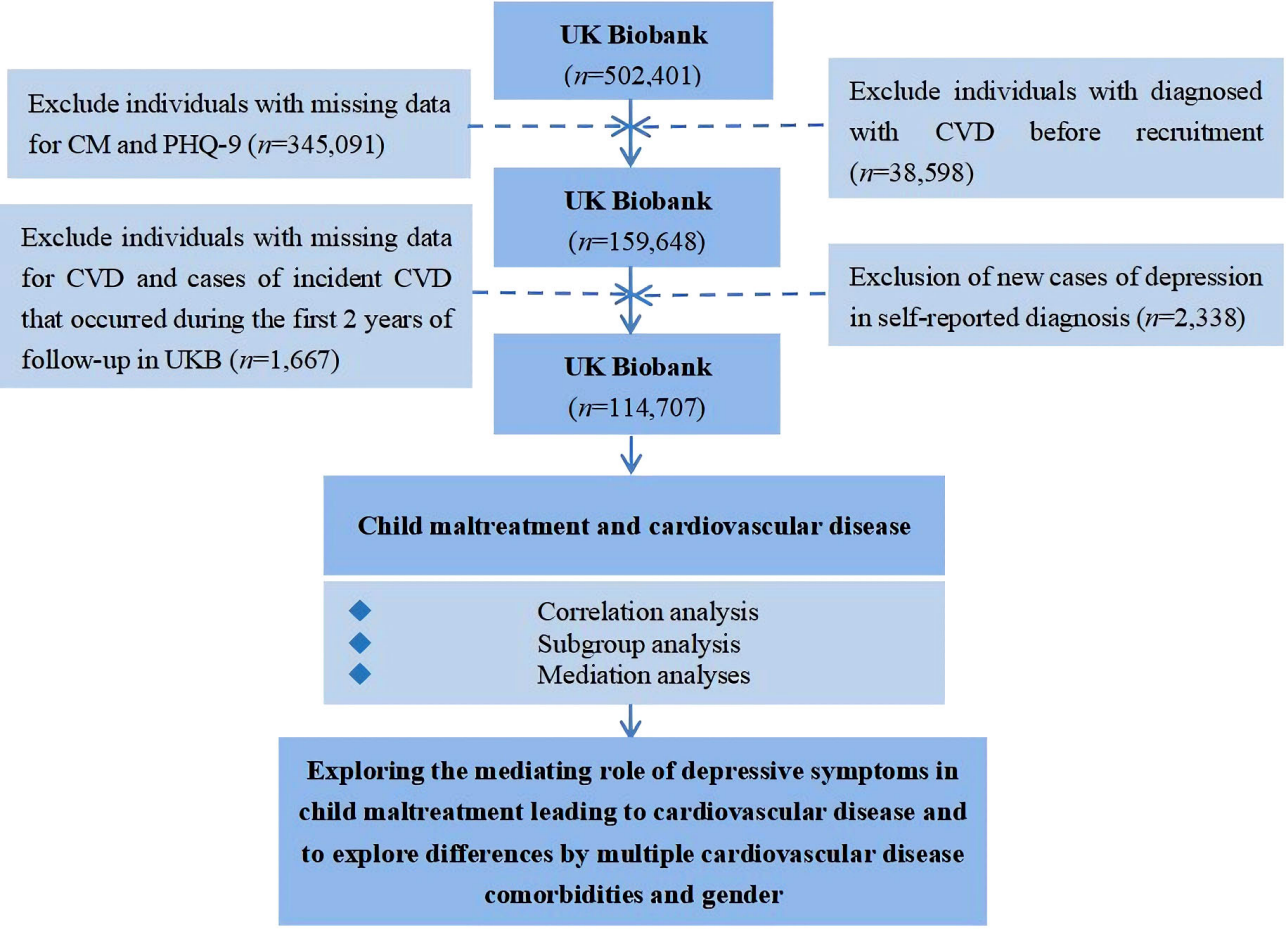
The flowchart for data processing and statistical analysis using the UK Biobank in the present work.

To investigate whether depressive symptoms mediate the relationship between CM and different CVDs, we established exclusion criteria for all participants: (1) participants with missing information in the CM and PHQ-9 scales; (2) to determine the number of new cases of outcome variables, patients diagnosed with CVD within 2 years after pre-recruitment diagnosis and follow-up were excluded (23); (3) To ensure the continuity of causality in exploring the mediating role, we consider it necessary to remove individuals diagnosed with depression from the self-report during recruitment, considering the collection of online questionnaires on psychological issues that started relatively late. Finally, we included 114,707 study participants (*n* = 16,142 for the CVD group and *n* = 98,565 for the non-CVD group), and the detailed process of participant selection is shown in **Figure 1**.

### 2.2 Ascertainment of CM

CM was assessed using the self-report CTS (24), a simplified version of the Childhood Trauma Questionnaire (CTQ) consisting of five items: physical abuse, physical neglect, emotional abuse, emotional neglect, and sexual abuse (pre-16 years). High correlations existed between the CTS items and the CTQ total score (r = 0.88) and corresponding dimensions (r = 0.55-0.87). Cronbach’s α indicated acceptable internal consistency (α=0.757) (25). A validation study showed Likert thresholds can classify presence/absence for each CM category. We reverse-scored physical and emotional neglect and then coded each domain as present/absent using those thresholds (15) (**Supplementary Table S1**). The exposure was the summed CM score (0–5).

### 2.3 Ascertainment of depression symptoms

Depression was measured using the PHQ-9. The PHQ-9 scores range from 0 to 27 (total score 0-4: minimal depression, 5-9: mild depression, 10-14: moderate depression, 15 -19: moderately severe depression, and 20-27: severe depression) (26), which was a continuous variable in subsequent analyses. **Supplementary Table S2** details the selection of variables in the scale and the collection of questionnaire content was done at baseline. To establish temporal precedence in the mediation model, individuals with a history of CVD or depression were excluded, ensuring that depressive symptoms could be evaluated as occurring prior to CVD onset.

### 2.4 Ascertainment of outcomes

Our definition of outcome is based on the International Statistical Classification of Diseases and Related Health Problems 10th Revision (ICD-10) (27) admission data, professionally diagnosed psychological issues, and self-reported diseases (non-cancer disease codes) from the UK Biobank, as shown in **Supplementary Table S3**. The time and reasons for admission in our study were obtained by linking to records from Health Event Statistics (England and Wales) and Scottish Morbidity Records (Scotland). Specific information can be found online (https://biobank.ctsu.ox.ac.uk/crystal/field.cgi?id=41270). The occurrence of CVD is defined as hypertension (HBP), heart failure (HF), coronary artery disease (CAD), and stroke, or at least one CVD has been reported among these four diseases. Given the high prevalence of HBP and its role in the early development of CVD, we believe this is a valid and meaningful result. It also includes cases of 2, 3, and 4 CVD comorbidities, all of which are defined as binary variables (0=non-disease, 1=diseased).

### 2.5 Ascertainment of confounders

Covariates (28) were selected at recruitment. And they included age (years), gender (0=female, 1=male), family history of CVD (0=no, 1=yes), ethnic background (1=European, 2=non-European), and qualifications (1= university, 2= below university, 3= others). We used maternal smoking during pregnancy (coded as 0 = no, 1 = yes) as a proxy for childhood socioeconomic position (SEP), given the absence of direct SEP measures in the sample. This approach is supported by evidence indicating that maternal smoking is consistently associated with lower socioeconomic status in multiple population settings, including those similar to the present study population (3). The Townsend Deprivation Index (TDI), a well-established area-level measure of socioeconomic disadvantage, was used as an indicator of early life SEP (3).

### 2.6 Statistical analysis

#### 2.6.1 Relationship between CM, depression symptoms, and CVD

First, to better understand the data distribution and characteristics, we described and tested the significance of differences in baseline characteristics and desired variables of participants under different gender subgroups. Continuous data are presented as means (standard deviations) and were tested using independent samples t-tests or Mann-Whitney U-tests. Categorical variables were presented as frequencies (percentages) and tested using chi-square tests.

Next, across the cohort, based on controlling for potential covariates, we assessed associations between CM scores and depression symptoms as well as different types of CVD using logistic models. To better explore whether there is a dose-response relationship between CM scores and CVD and to further understand the results revealed by the model, we used two separate approaches to interpret the results. First, the type of CM experienced by each participant was calculated (0–5 scores were divided into six separate groups), modeled, and the odds ratio (OR) was calculated. In addition, we calculated the marginal effects of CM scores on depression and CVD (29, 30) to quantify the amount of change in risk for each disease in logistic regressions.

#### 2.6.2 Subgroup analysis

To explore the potential modifying effects of categorical variables between CM and CVD, we conducted several subgroup analyses by gender (female/male), ethnic background (European/non-European), qualifications (university, below university, or others), maternal smoking around birth (no/yes), and family history (no/yes).

#### 2.6.3 Mediation analysis from CM to depression symptoms and CVD

Finally, we applied mediation analyses to explore whether depression symptoms have an indirect role in the association between the CM scores and single CVD. Considering that single CVD usually occurs together (19), we also performed mediation analyses with multiple CVD comorbidities as outcome variables. We also explored the size of the proportions mediated by depressive symptoms in different gender groups. Additionally, in the exposure-mediator model (CM scores to depression symptoms), we used a linear model, whereas in the mediator-outcome model (from depression symptoms to different types of CVD), we used a logistic model. The mediated proportions were calculated using closed-form parametric function estimation and bootstrapping in the “CMAverse” software package (31). We used the CMAverse package in R for causal mediation analysis, as it allows estimation of natural direct and indirect effects under appropriate assumptions and accommodates complex survey designs and mixed model types (e.g., linear for the mediator and logistic for the outcome). This approach provides a flexible and robust framework for mediation analysis in epidemiological studies.

All the above analyses were performed after multiple imputation (MI) (32). We set m = 5 imputed datasets to cope with moderate levels of missing data. The estimation model included all variables used in the main analyses (e.g., childhood maltreatment scores, depressive symptoms, cardiovascular disease status, age, gender, and sociodemographic covariates) to maintain the structure of associations between variables. Model convergence was confirmed by observing the stability of parameter estimates during iterations. The level of statistical significance was 95%, two-sided (P ≤ 0.05), and all of the above analyses were performed in R software (R 2.4.1).

## 3 Results

### 3.1 Baseline characteristics

Gender differences in baseline characteristics for the 114,707 UK Biobank participants are summarized in **Table 1**. Female exhibited higher levels of CM score and depressive symptoms, as well as greater prevalence of HBP and any CVD, while male showed higher rates of HF, coronary artery disease, and stroke. Female were also more likely to report maternal smoking during pregnancy, family history of CVD, and socioeconomic deprivation—factors that may contribute to differential cardiovascular risk trajectories by gender.

**Table 1:** Characteristics of participants graded according to gender in the UK Biobank.

Characteristics	Level	Participants, No. (%)			*P* value
		Total (N = 114707)	Female (N = 69103)	Male (N = 45604)	
Age (Mean ± SD)		54.89 (7.76)	54.67 (7.65)	55.24 (7.91)	<0.001
TDI (Mean ± SD)		-1.71 (2.82)	-1.67 (2.82)	-1.77 (2.83)	5.92×10^-13^
Maternal smoking around birth (%)	No	86161 (75.11)	51961 (75.19)	34200 (74.99)	0.453
	Yes	28546 (24.89)	17142 (24.81)	11404 (25.01)	
Ethnic background (%)	European	111050 (96.81)	66953 (96.89)	44097 (96.70)	0.071
	Non-European	3657 (3.19)	2150 (3.11)	1507 (3.30)	
Family history (%)	No	48164 (41.99)	27680 (40.06)	20484 (44.92)	<0.001
	Yes	66543 (58.01)	41423 (59.94)	25120 (55.08)	
Qualifications (%)	University	54183 (47.24)	31422 (45.47)	22761 (49.91)	<0.001
	Below university	47864 (41.73)	29504 (42.70)	18360 (40.26)	
	Others	12660 (11.04)	8177 (11.83)	4483 (9.83)	
CM scores (Mean ± SD)		1.537 (0.92)	1.583 (0.98)	1.466 (0.83)	<0.001
Depression symptoms (Mean ± SD)		2.688 (3.59)	2.938 (3.71)	2.308 (3.37)	<0.001
HBP (%)	No	101926 (88.86)	62482 (90.42)	39444 (86.49)	<0.001
	Yes	12781 (11.14)	6621 (9.58)	6160 (13.51)	
HF (%)	No	113471 (98.92)	68598 (99.27)	44873 (98.40)	<0.001
	Yes	1236 (1.08)	505 (0.73)	731 (1.60)	
CAD (%)	No	110272 (96.13)	67368 (97.49)	42904 (94.08)	<0.001
	Yes	4435 (3.87)	1735 (2.51)	2700 (5.92)	
Stroke (%)	No	113360 (98.83)	68465 (99.08)	44895 (98.45)	<0.001
	Yes	1347 (1.17)	638 (0.92)	709 (1.55)	
At least one CVD (%)	No	98565 (85.93)	61007 (88.28)	37558 (82.36)	<0.001
	Yes	16142 (14.07)	8096 (11.72)	8046 (17.64)	

### 3.2 Relationship between CM, depression symptoms, and different types of CVD

#### 3.2.1 Estimated relation between CM, depression symptoms, and single or at least one CVD

The results of correlation tests between CM scores and single or at least one CVD, and depression symptoms in all cohorts are presented in **Table 2**. It showed that there were generally significant differences between these factors. Notably, in terms of CM scores, with the strongest correlation with CAD (odds ratios [OR]=1.10, 95% confidence interval [CI]: 1.06~1.14, *P* = 1.72×10^-8^), whereas stroke (OR = 0.99, 95% CI: 0.93~1.06, *P* = 0.817) showed no correlation. In terms of depression symptoms, the correlations with CVD were all significant, with the strongest correlation with HF in particular (OR = 1.21, 95% CI: 1.14~1.27, *P* = 3.44×10^-12^). In the correlation study of different gender groups, the results shown in **Supplementary Table S4** can be observed. Interestingly, the correlation between CM scores and CVD seems to be stronger in females compared to males.

**Table 2:** Association of CM with single or at least one CVD and depressive symptoms by using a logistic regression model in all cohorts (odd ratios and 95% CIs).

Outcomes	CM scores OR (95%CI)	*P* value	Depression symptoms OR (95%CI)	*P* value
HBP	1.03(1.01~1.06)	0.001	1.19(1.16~1.21)	<0.001
HF	1.09(1.02~1.16)	0.005	1.21(1.14~1.27)	3.44×10^-12^
CAD	1.10(1.06~1.14)	1.72×10^-8^	1.18(1.15~1.22)	<0.001
Stroke	0.99(0.93~1.06)	0.817	1.16(1.10~1.22)	3.35×10^-8^
At least one	1.05(1.03~1.07)	2.15×10^-6^	1.19(1.17~1.21)	<0.001

HBP, hypertension; HF, heart failure; CAD, coronary artery disease; CVD, cardiovascular diseases; CM, childhood maltreatment.

**Supplementary Figure S1** demonstrates the dose-response relationship between different levels of CM scores and the outcome variables after controlling for confounders. Overall, a progressive increase in the prevalence of HBP, CAD, and at least one CVD was observed with higher CM scores. Interestingly, with the highest odd ratios for CAD (OR = 3.12, 95% CI: 2.15~4.51, *P* < 0.001) with a CM score of 5. **Supplementary Figures S2**, **3** shows the dose-response relationship between different levels of CM scores and single or at least one CVD in different gender groups.

In all cohorts, marginal effect analyses showed that, after adjusting for key confounders, higher CM scores were associated with increased probabilities of HBP, heart failure, coronary artery disease, any CVD, and depressive symptoms (**Supplementary Table S5**). Each one-unit increase in CM score was linked to a 1.03% higher absolute risk of having at least one CVD and a substantial 2.38% increase in the likelihood of depressive symptoms. The association with stroke was not statistically significant (*P* = 0.341), suggesting a weaker or absent link in this outcome.

#### 3.2.2 Estimated relation between CM, depression symptoms, and multiple CVD comorbidities

The results of the study on the correlation of CM scores or depression symptoms with different multiple CVD comorbidities are detailed in **Supplementary Table S6**. Overall, a varying number of CVD comorbidities were associated with a slightly higher risk of developing CM and depressive symptoms compared with individual CVDs, but the results were not entirely significant. Of these, CM scores were most strongly associated with CAD and HF comorbidities (OR = 1.13, 95% CI: 1.04~1.23, *P* = 0.004), whereas depressive symptoms were most strongly associated with the 4 CVD comorbidities (OR = 1.64, 95% CI: 1.29~2.10, *P* = 6.20×10^-5^).

Besides, the dose-response relationship between different levels of CM scores and multiple CVD comorbidities is shown in **Supplementary Table S7**. The results showed a dose-response relationship between most CVD comorbidities and CM scores, although the results were not all significant.

### 3.3 Results of subgroup analysis

Subgroup analyses (**Supplementary Tables S8**–**S10**) revealed that the association between CM and CVD varied across key demographic and familial factors. Notably, the effect was stronger among women, individuals with a family history of CVD, and those exposed to maternal smoking around birth, suggesting that early-life and familial vulnerabilities may amplify the long-term cardiovascular impact of CM.

### 3.4 Mediating pathways between CM and different types of CVD

#### 3.4.1 Total effect of CM on CVD (path c)

**Figure 2:**
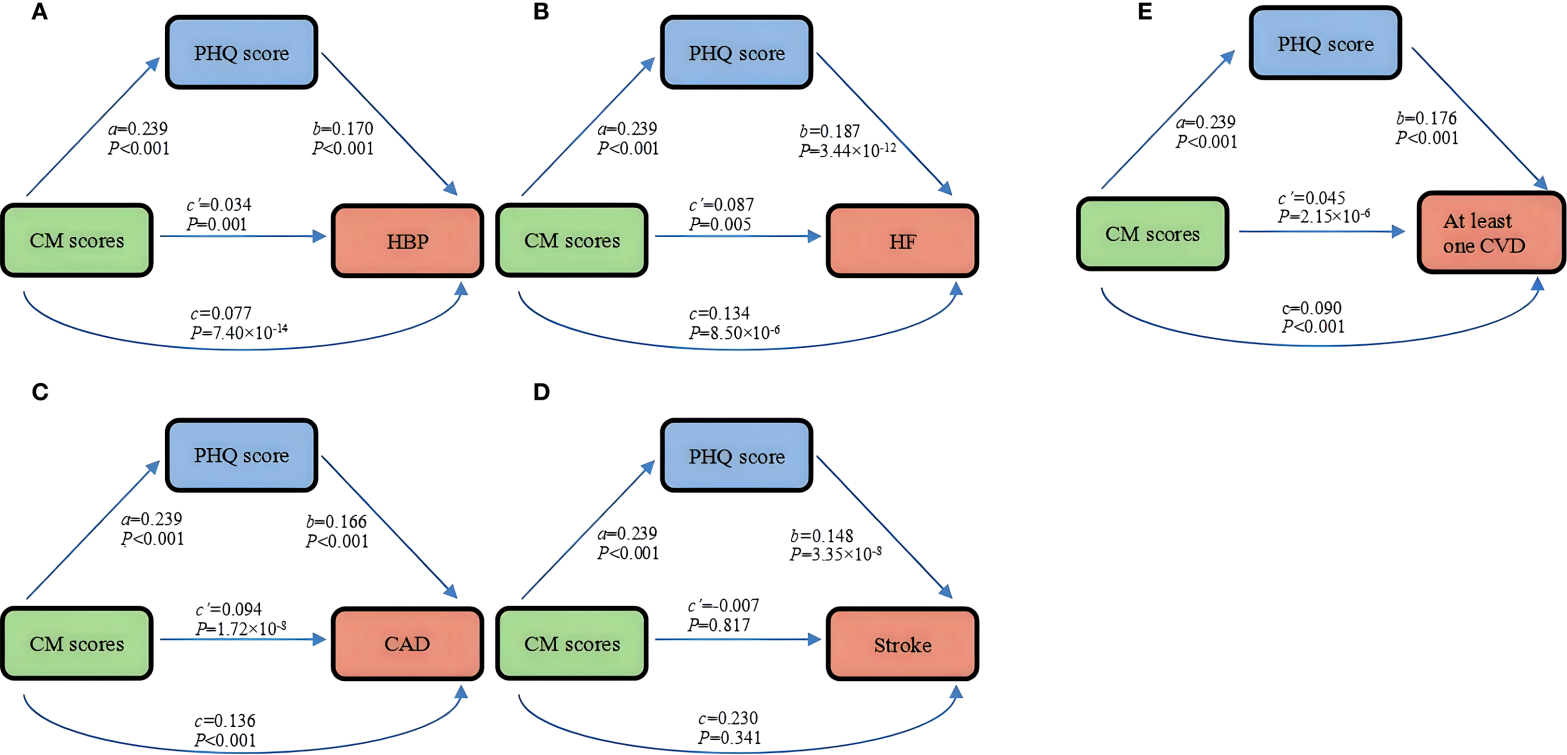
Mediation effect of depressive symptoms in the association between CM and single or at least one CVD in all cohorts: **(A)** hypertension, **(B)** heart failure, **(C)** at least one CVD, **(D)** coronary artery disease, **(E)** stroke. HBP, hypertension; HF, heart failure; CAD, coronary artery disease; CVD, cardiovascular diseases; CM, childhood maltreatment; PHQ-9, Patient Health Questionnaire 9-item.

Here, we sought to test the total effect of CM and CVD in mediation analysis. After adjusting for potential covariates, we observed that compared to other single CVDs, CM scores had the greatest effect on having CAD (OR = 1.13, 95% CI: 1.10~1.17, *P* < 0.001) among all participants (**Figure 2**). However, we did not find a significant association in stroke (*P* = 0.341). Subsequently, when evaluating the correlation between CM and multiple CVD comorbidities, some results are significant. Especially, the CM score has the strongest correlation with comorbidities of HF and CAD (OR = 1.16, 95% CI: 1.07~1.24) (**Supplementary Figures S4**, **5**). Among females, we observed the most significant association between CM scores and CAD (OR = 1.19, 95% CI: 1.15~1.24), however, among males, the effect of CM scores on HF (OR = 1.13, 95% CI: 1.05~1.22) was greater (**Supplementary Figures S6**, **7**).

#### 3.4.2 Effect of CM on depressive symptoms and effect of depressive symptoms on CVD (paths a and b)

Additionally, CM scores associated with higher depressive symptoms had similar effect sizes across all cohorts and gender groups (*β* = 0.239 in all cohorts and the male group, and *β* = 0.240 in the female group) (**Supplementary Figures S4**–**7**).

We further found that the ORs for the positive effect of depressive symptoms on a single or at least one CVD ranged from 1.16 to 1.21 (**Figure 2**.). At the overall level, the correlation coefficient between depressive symptoms and comorbidities with different numbers of CVDs is greater than that of a single CVD, with depression symptoms having the greatest impact on patients with four CVD comorbidities (OR = 1.64, 95% CI: 1.29~2.10) (**Supplementary Figures S4**, **5**). Interestingly, the correlation between depressive symptoms and HF was slightly stronger in females (OR = 1.33, 95% CI:1.24-1.43), whereas the correlation between depressive symptoms and at least one CVD was slightly stronger in males (OR = 1.17, 95% CI:1.14~1.20) (**Supplementary Figures S6**, **7**).

#### 3.4.3 The ratio of the mediation effect to the total effect

In the following two tables (**Tables 3**, **4**) we only present results where mediating effects are present and significant. Specifically, the mediating effect of depressive symptoms accounted for approximately 31.03%~55.28% of the total effect in the full cohort with a single CVD as the outcome (**Table 3**), and approximately 28.91%~55.02% (**Table 4**) in the different gender groups. However, it accounted for approximately 22.93%~36.46% in the full cohort with multiple CVD comorbidities as outcomes (**Table 3**). It is important to emphasize that we did not calculate here the proportion of mediators whose mediation effect was opposite in sign to the total effect and whose outcome was not significant, because such ratios are not meaningful in this case.

**Table 3:** Comparison of the proportion of depressive symptoms mediating the relationship between CM and different amounts of CVD.

Amounts of CVD	Outcomes	Mediation proportion	*P* value
Single CVD	HBP	0.55(0.39~0.71)	5.00×10^-12^
	HF	0.35(0.17~0.53)	0.001
	CAD	0.31(0.22~0.39)	6.17×10^-12^
	Any one CVD	0.49(0.38~0.60)	<0.001
Multiple CVD comorbidities	HBP+CAD	0.36(0.21~0.51)	1.38×10^-6^
	HF+CAD	0.23(0.06~0.39)	0.006
	HBP+HF+CAD	0.34(0.19~0.49)	0.036

HBP, hypertension; HF, heart failure; CAD, coronary artery disease; CVD, cardiovascular diseases; CM, childhood maltreatment.

**Table 4:** Comparison of the proportion of depressive symptoms mediating the relationship between CM and single CVD or at least one in different gender groups.

Gender	Outcomes	Mediation proportion	*P* value
Female	HBP	0.55(0.37~0.73)	3.37×10^-9^
	CAD	0.29(0.20~0.37)	2.37×10^-11^
	Any one CVD	0.47(0.35~0.59)	6.88×10^-15^
Male	CAD	0.38(0.13~0.62)	0.002
	Any one CVD	0.55(0.30~0.79)	1.19×10^-5^

HBP, hypertension; HF, heart failure; CAD, coronary artery disease; CVD, cardiovascular diseases; CM, childhood maltreatment.

## 4 Discussion

### 4.1 Summary and comparison with existing studies

#### 4.1.1 Effects of CM on CVD and depression symptoms

In the present study, we found that after controlling for childhood socioeconomic and demographic factors, CM scores led to a higher risk of depressive symptoms and increased the risk of different types of CVD. In addition, CM scores showed a dose-response relationship with CVD (especially in females and comorbidities) and the marginal effect is significant (except for stroke where the results were not significant). The mediation proportions (22.93%~55.28%) suggest that depressive symptoms play a meaningful role in the pathway from childhood maltreatment to CVD, with potential implications for prevention—addressing depression may help reduce a substantial fraction of CVD risk in this population. In particular, we found that the mediation ratio for depressive symptoms varied by gender.

Our findings are consistent with previous population-based, long-term studies conducted in different countries that found a significant correlation between CM scores and depressive symptoms or CVD (33). Furthermore, we also found a dose-response relationship between CM and CVD, which is similar to previous studies (7). Notably, CM is more closely associated with CVD comorbidities compared to individual CVD, a result that is explained by the fact that the biological and inflammatory system responses brought about by CM may be associated with the manifestation of multiple CVD diseases. In addition, the results of this study showed a higher correlation between CM and CVD in female than in male, and we consider that it may be possible that CM types such as sexual abuse and physical neglect may be more common in female. And results from previous studies of CM populations have also shown a higher risk of CAD (but not limited to CAD) in female than in male (3). However, the findings from the current review of the literature are not unique in terms of definitive conclusions regarding gender differences (34).

Negative results for stroke and CM or depression symptoms in association studies may either be due to a lack of statistical efficacy because of the low incidence of stroke events or suggest that stroke may develop through pathophysiologic mechanisms different from those of other CVD, such as a weaker influence of psychosocial stress pathways on stroke or its onset being driven more by an acute vascular event or genetic factors.

#### 4.1.2 Mediated regulation and potential impact mechanisms

We interpret these findings as compared to a single CVD, the depression symptoms explained to varying degrees the correlation between CM score and CVD comorbidities (21.43%~34.21%). We consider that the reason for this result may be due to the diversity of multiple CVD comorbidities, which may result in other pathological, physiological (inflammation, blood lipids, etc.), and neurobiological factors playing a more significant role, thus masking the mediating effect of some depression (35). However, in males, the mediating proportion of depressive symptoms between CM and CVD is higher, suggesting potential sex-specific pathways in the psychological sequelae of childhood maltreatment. Combined with the previous discussion on gender differences between CM and CVD, there may be other common factors that play a mediating role, which may require further research (36). Psychosocial factors may play a smaller role in stroke or its onset. These gender differences may reflect, in part, higher stress sensitivity and greater likelihood of internalizing responses among females, which is also congruent with recent evidence on behavioral pathways to cardiovascular risk (37). Because the total and direct effects between CM scores and some CVDs (e.g., stroke, HF, etc.) were not significant, we hypothesized that there was no mediating effect. Although we adjusted for a range of pre-exposure and time-varying covariates—including age, sex, socioeconomic status, childhood home environment, and parental history of CVD—residual confounding cannot be fully ruled out. While these adjustments help strengthen the validity of the estimated mediation pathways, the possibility remains that underreporting of diseases or lack of corroborating evidence may affect the findings. Therefore, establishing triple sequential causality is essential for confirming these associations.

In summary, depressive symptoms significantly mediated the association between CM and CVD, consistent with previous findings (38). The above findings are consistent with a potential role of psychological factors in the association between CM and CVD. Specifically, persistent and intense stress early in life may reactively alter the HPA, leading to varying degrees of mood disorders and elevated or decreased cortisol concentrations, which can lead to the development of depression or other psychological problems (39); autonomous alterations in the nervous system, platelet receptors, coagulation, altered endothelial function, and several other mechanisms may link depressive symptoms to CVD (14, 35). However, no mediating role of depression was observed in the longitudinal pathway analysis of American adolescents/adults, indicating that the mediating role of depression needs further research (17). The potential common mechanism between CM and CVD may also stem from individual or common physiological and behavioral factors (40), providing new clues for the causal relationship between the two and requiring further exploration and confirmation.

### 4.2 Strengths and limitations

Our work has several advantages over previous studies. First, considering that an unrepresentative sample in a small sample may increase sampling error, this study used a large cohort based on a mature European population to increase the accuracy and breadth of the results. Second, CM-related findings provide information that is not limited to one form; but also includes marginal effects of relative risk indicators OR and absolute risk differences (30). Third, in further mediation analyses, after exploring the indirect effects of psychological factors in the association of CM with CVD, we sequentially examined gender differences and comorbidity differences, which will help us to target the prevention of CVD by understanding the relative contribution of depression, thereby reducing the risk of future disease and improving quality of life. Finally, we included childhood SEP as a confounder in correlation tests and mediation analyses to increase the intrinsic validity and confidence of the results.

Our study also has limitations. First, in terms of variable selection, we assessed CM using retrospective measures, which may be affected by measurement and recall bias (41). For example, individuals with current mental or physical health conditions may be more likely to recall or emphasize past adverse experiences. This may inflate the observed associations between CM and later outcomes such as depression or cardiovascular disease, potentially introducing upward bias. Conversely, underreporting due to repression or normalization of trauma could attenuate associations; confounders such as marital status, biomarkers, and relationship with parents were not considered, which may have affected our results (5). Due to the observational design, residual confounding and reverse causality cannot be fully ruled out, and findings should be interpreted with caution. Given the small sample sizes for individual CVDs such as heart failure and stroke, the significant differences in test results may indicate insufficient validity of the analyses. While this exclusion may introduce healthy-participant bias, it strengthens the validity of the assumed causal sequence in our mediation analysis.

## 5 Conclusions

CM was associated with higher depressive symptoms and higher CVD risk. Mediation analyses indicated that depressive symptoms mediated part of the association, with proportions varying by gender and by comorbidity pattern. These findings highlight the value of addressing depressive symptoms in adults with CM histories as a potential means to reduce CVD risk.

## Data Availability

The original contributions presented in the study are included in the article/**Supplementary Material**. Further inquiries can be directed to the corresponding author.
