# Supplementary material for: Uncovering psychologically mediated pathways to cardiovascular diseases: depressive symptoms as a mediator between childhood maltreatment and single or multiple cardiovascular disease comorbidities
Source: Front Psychiatry. 2025 Oct 9;16:1560961. doi: 10.3389/fpsyt.2025.1560961 (PMC12550589; doi:10.3389/fpsyt.2025.1560961)
Supplement: Supplementary file 1 [file Supplementaryfile1.docx]

Supplementary Material

# 1 Supplementary Data

# 2 Supplementary Figures and Tables

# 2.1 Supplementary Tables

Table S1 Determination of child maltreatments in the UK Biobank database based on the Childhood Trauma Screener – 5 items (CTS-5).

| CM | Prefer not to answer | Never true | Rarely true | Sometimes true | Often true | Very often true |
| --- | --- | --- | --- | --- | --- | --- |
| Physical abuse: Physically abused by family as a child | 358(0.2) | 127,171(80.1) | 16,839(10.7) | 10,609(6.7) | 1,422(0.9) | 911(0.5) |
| Emotional abuse: Felt hated by family members as a child | 454(0.2) | 132,323(84.1) | 9,538(6.1) | 10,426(6.6) | 2,500(1.6) | 2,069(1.3) |
| Sexual abuse: Sexually molested as a child | 1,852(1.2) | 141,818(90.1) | 7,183(4.6) | 4,999(3.2) | 797(5.1) | 661(4.2) |
| Physical neglect: Someone to take to the doctor when needed as a child | 1,074(0.7) | 3,376(2.1) | 1,305(0.8) | 4,316(2.7) | 16,718(10.6) | 130,521(83.0) |
| Emotional neglect: Felt loved as a child | 628(0.4) | 2,307(1.5) | 7,319(4.7) | 25,653(16.3) | 39,841(25.3) | 81,562(51.8) |

Note: The numbers presented are n (%). Red fonts indicate the response that is categorized as child maltreatment.

Table S2 Determination of depressive symptoms in the UK Biobank database based on the Patient Health Questionnaire 9-item (PHQ-9 scale).

| PHQ-9 | UK Biobank | Code |  |
| --- | --- | --- | --- |
| D.1 Anhedonia | Recent lack of interest or pleasure in doing things | 20514.0.0 | Range 0-3, > 0 Endorsed |
| D.2 Depressed mood | Recent feelings of depression | 20510.0.0 |  |
| D.3 Sleep problems | Trouble falling or staying asleep, or sleeping too much | 20517.0.0 |  |
| D.4 Fatigue | Recent feelings of tiredness or low energy | 20519.0.0 |  |
| D.5 Appetite changes | Recent poor appetite or overeating | 20511.0.0 |  |
| D.6 Feelings of inadequacy | Recent feelings of inadequacy | 20507.0.0 |  |
| D.7 Cognitive problems | Recent trouble concentrating on things | 20508.0.0 |  |
| D.8 Psychomotor changes | Recent changes in speed/amount of moving or speaking | 20518.0.0 |  |
| D.9 Suicidal ideation | Recent thoughts of suicide or self-harm | 20513.0.0 |  |

Note: The PHQ-9 is validated as a depressive symptom severity measure (total score 0–4: minimal depression, 5–9: mild depression, 10–14: moderate depression, 15–19: moderately severe depression, and 20–27: severe depression).

Table S3 Determination of the diagnosis of four cardiovascular diseases in the UK Biobank database according to ICD-10, professionally diagnosed psychological issues, and self-reported diseases.

| Disease | After baseline |
| --- | --- |
| Hypertension | 6150, 20002  ICD-10: I10, I11, I12, I13, I15 |
| Coronary artery disease | 6150, 20002  ICD-10: I20, I21, I22, I23, I24, I25 |
| Stroke | 6150, 20002  ICD-10: I60, I61, I62, I63, I64, I69 |
| Heart failure | 6150, 20002  ICD-10: I50 |

Table S4 Association of CM with single or at least one CVD and depressive symptoms by using a logistic regression model in different gender groups (odd ratios and 95% CIs).

| Outcomes | Female | | | | Male | | | |
| --- | --- | --- | --- | --- | --- | --- | --- | --- |
|  | CM scores  OR (95%CI) | *P* value | Depressive symptom  OR (95%CI) | *P* value | CM scores  OR (95%CI) | *P* value | Depressive symptom  OR (95%CI) | *P* value |
| HBP | 1.04(1.01~1.07) | 0.005 | 1.20(1.17~1.23) | <0.001 | 1.03(0.99~1.06) | 0.105 | 1.17(1.14~1.20) | <0.001 |
| HF | 1.06(0.97~1.15) | 0.218 | 1.33(1.24~1.43) | 1.03×10^-15^ | 1.12(1.03~1.22) | 0.007 | 1.09(1.00~1.18) | 0.044 |
| CAD | 1.15(1.10~1.20) | 4.38×10^-9^ | 1.23(1.19~1.29) | <0.001 | 1.05(1.00~1.10) | 0.034 | 1.13(1.09~1.18) | 4.02×10^-9^ |
| Stroke | 1.00(0.92~1.09) | 0.998 | 1.19(1.11~1.27) | 1.77×10^-6^ | 0.98(0.89~1.08) | 0.685 | 1.13(1.04~1.22) | 0.003 |
| At least one | 1.06(1.03~1.08) | 1.08×10^-5^ | 1.21(1.19~1.24) | <0.001 | 1.03(1.00~1.06) | 0.039 | 1.17(1.14~1.20) | <0.001 |

Abbreviation: HBP hypertension, HF heart failure, CAD coronary artery disease, CVD cardiovascular diseases, CM child maltreatment.

Table S5 The marginal effect of CM with single or at least one CVD and depressive symptoms in all populations.

| Outcomes | *AME*, %(95%CI) | *SE* | *Z* value | *P* value |
| --- | --- | --- | --- | --- |
| HBP | 0.73(0.54~0.92) | 0.001 | 7.440 | <0.001 |
| HF | 0.14(0.08~0.20) | <0.001 | 4.422 | <0.001 |
| CAD | 0.50(0.38~0.61) | 0.001 | 8.384 | <0.001 |
| Stroke | 0.03(-0.04~0.10) | 0.004 | 0.952 | 0.341 |
| At least one CVD | 1.03(0.82~1.24) | 0.001 | 9.582 | <0.001 |
| depression symptoms | 2.38(2.28~2.48) | 0.001 | 44.377 | <0.001 |

Abbreviation: HBP hypertension, HF heart failure, CAD coronary artery disease, CVD cardiovascular diseases, CM child maltreatment.

Table S6 Association of CM with different multiple CVD comorbidities and depressive symptoms by using a logistic regression model in all cohorts (odd ratios and 95% CIs).

| Outcomes | CM scores  OR (95%CI) | *P* value | Depressive symptoms  OR (95%CI) | *P* value |
| --- | --- | --- | --- | --- |
| HBP+HF | 1.06(0.98~1.15) | 0.141 | 1.25(1.16~1.34) | 1.29×10^-9^ |
| HBP+CAD | 1.08(1.03~1.13) | 0.001 | 1.21(1.16~1.26) | <0.001 |
| HBP+Stroke | 1.02(0.93~1.11) | 0.710 | 1.19(1.10~1.28) | 1.40×10^-5^ |
| HF+CAD | 1.13(1.04~1.23) | 0.004 | 1.15(1.07~1.24) | 0.002 |
| HF+Stroke | 0.91(0.70~1.19) | 0.487 | 1.41(1.17~1.70) | 1.24×10^-6^ |
| CAD+Stroke | 1.07(0.91~1.26) | 0.406 | 1.24(1.07~1.43) | 0.005 |
| HBP+HF+CAD | 1.08(1.01~1.15) | 0.017 | 1.17(1.06~1.30) | 0.002 |
| HBP+HF+Stroke | 1.01(0.75~1.35) | 0.966 | 1.60(1.31~1.95) | 3.36×10^-6^ |
| HBP+CAD+Stroke | 1.11(0.91~1.35) | 0.285 | 1.30(1.10~1.55) | 0.002 |
| HF+CAD+Stroke | 0.90(0.64~1.27) | 0.560 | 1.45(1.16~1.83) | 0.001 |
| HBP+HF+CAD+Stroke | 0.93(0.63~1.39) | 0.723 | 1.64(1.29~2.10) | 6.20×10^-5^ |

Abbreviation: HBP hypertension, HF heart failure, CAD coronary artery disease, CVD cardiovascular diseases, CM childhood maltreatment.

Table S7 Dose-response relationships between CM scores and multiple CVD comorbidities in all population after adjustment for confounding variables (odd ratios and 95% CIs).

| Outcomes | CM scores | | | | | | | | | | |
| --- | --- | --- | --- | --- | --- | --- | --- | --- | --- | --- | --- |
|  | 0 | 1  OR (95%CI) | *P* value | 2  OR (95%CI) | *P* value | 3  OR (95%CI) | *P* value | 4  OR (95%CI) | *P* value | 5  OR (95%CI) | *P* value |
| HBP+HF | Ref. | 1.23(1.01-1.49) | 0.035 | 1.37(1.03-1.82) | 0.031 | 1.37(0.89-12.12) | 0.153 | 1.18(0.56-2.52) | 0.662 | 2.25(0.71-4.08) | 0.179 |
| HBP+CAD | Ref. | 1.10(0.98-1.22) | 0.097 | 1.26(1.07-1.48) | 0.097 | 1.17(0.91-1.51) | 0.226 | 2.15(1.58-2.93) | <0.001 | 3.14(1.86-5.33) | <0.001 |
| HBP+Stroke | Ref. | 1.08(0.89-1.32) | 0.429 | 0.98(0.71-1.35) | 0.893 | 1.05(0.65-1.70) | 0.813 | 1.10(0.52-2.34) | 0.806 | 3.91(1.72-8.89) | <0.001 |
| HF+CAD | Ref. | 1.18(0.98-1.43) | 0.083 | 1.33(1.00-1.77) | 0.047 | 1.44(0.95-2.18) | 0.086 | 2.49(1.48-4.21) | <0.001 | 1.42(0.35-5.74) | 0.640 |
| HF+Stroke | Ref. | 0.83(0.44-1.56) | 0.556 | 1.39(0.63-3.08) | 0.420 | 0.49(0.07-3.58) | 0.483 | 0.00(0.00-Inf) | 0.984 | 5.45(0.74-10.19) | 0.096 |
| CAD+Stroke | Ref. | 0.89(0.59-1.35) | 0.591 | 1.54(0.91-2.62) | 0.108 | 0.50(0.12-2.02) | 0.324 | 1.37(0.34-5.60) | 0.660 | 9.26(2.87-29.85) | <0.001 |
| HBP+HF+CAD | Ref. | 1.15(0.89-1.48) | 0.300 | 1.44(1.00-2.07) | 0.048 | 1.07(0.57-2.03) | 0.829 | 1.50(0.61-3.66) | 0.377 | 2.67(0.66-10.86) | 0.179 |
| HBP+HF  +Stroke | Ref. | 1.15(0.54-2.46) | 0.716 | 1.82(0.70-4.76) | 0.223 | 0.89(0.12-6.58) | 0.908 | 0.00(0.00-Inf) | 0.990 | 9.52(1.26-17.85) | <0.001 |
| HBP+CAD  +Stroke | Ref. | 0.96(0.57-1.62) | 0.871 | 1.44(0.71-2.90) | 0.307 | 0.83(0.20-3.40) | 0.786 | 2.27(0.55-9.39) | 0.257 | 10.04(2.39-18.11) | 0.001 |
| HF+CAD  +Stroke | Ref. | 0.39(0.14-1.11) | 0.077 | 1.82(0.76-4.35) | 0.178 | 0.00(0.00-Inf) | 0.984 | 0.00(0.00-Inf) | 0.990 | 10.14(1.35-25.91) | 0.024 |
| HBP+HF+CAD+Stroke | Ref. | 0.32(0.07-1.35) | 0.120 | 1.94(0.66-5.67) | 0.225 | 0.00(0.00-Inf) | 0.989 | 0.00(0.00-Inf) | 0.993 | 16.21(2.11-30.62) | 0.007 |

Abbreviation: HBP hypertension, HF heart failure, CAD coronary artery disease, CVD cardiovascular diseases, CM child maltreatment.

Table S8 Effect size (stratified by categorical covariates) of CM on a single or at least one CVD in full cohort (odd ratios and 95% CIs).

| Subgroup | HBP  OR (95%CI) | *P*  value | HF  OR (95%CI) | *P*  value | CAD  OR (95%CI) | *P* value | Stroke  OR (95%CI) | *P* value | At least one  OR (95%CI) | *P*  value |
| --- | --- | --- | --- | --- | --- | --- | --- | --- | --- | --- |
| Maternal smoking around birth |  |  |  |  |  |  |  |  |  |  |
| No | 1.07(1.04~  1.09) | <0.001 | 1.15(1.07~  1.23) | <0.001 | 1.11(1.07~  1.15) | <0.001 | 1.03(0.96~  1.11) | 0.367 | 1.08(1.05~  1.10) | <0.001 |
| Yes | 1.10(1.07~  1.14) | <0.001 | 1.12(1.01~  1.24) | 0.030 | 1.21(1.15~  1.28) | <0.001 | 1.02(0.91~  1.13) | 0.725 | 1.13(1.09~  1.16) | <0.001 |
| Family history |  |  |  |  |  |  |  |  |  |  |
| No | 1.09(1.05~  1.12) | <0.001 | 1.17(1.07~  1.28) | 0.001 | 1.18(1.12~  1.23) | <0.001 | 1.07(0.97~  1.17) | 0.161 | 1.10(1.07~  1.14) | <0.001 |
| Yes | 1.08(1.06~  1.11) | <0.001 | 1.13(1.05~  1.22) | 0.002 | 1.13(1.09~  1.18) | <0.001 | 1.01(0.93~  1.10) | 0.770 | 1.10(1.07~  1.12) | <0.001 |
| Qualifications |  |  |  |  |  |  |  |  |  |  |
| University | 1.10(1.06~  1.13) | <0.001 | 1.17(1.06~  1.29) | 0.001 | 1.13(1.07~  1.19) | <0.001 | 1.10(1.00~  1.21) | 0.036 | 1.10(1.07~  1.14) | <0.001 |
| Below university | 1.06(1.02~  1.09) | <0.001 | 1.15(1.05~  1.26) | 0.002 | 1.15(1.09~  1.20) | <0.001 | 1.00(0.90~  1.09) | 0.928 | 1.08(1.05~  1.11) | <0.001 |
| Others | 1.14(1.08~  1.19) | <0.001 | 1.09(0.94~  1.24) | 0.230 | 1.20(1.12~  1.29) | <0.001 | 0.97(0.83~  1.13) | 0.748 | 1.14(1.09~  1.19) | <0.001 |
| Gender |  |  |  |  |  |  |  |  |  |  |
| Female | 1.09(1.07~  1.12) | <0.001 | 1.15(1.06~  1.25) | 0.001 | 1.22(1.16~  1.27) | <0.001 | 1.05(0.97~  1.14) | 0.198 | 1.12(1.09~  1.14) | <0.001 |
| Male | 1.07(1.04~  1.11) | <0.001 | 1.15(1.05~  1.25) | 0.001 | 1.09(1.04~  1.14) | <0.001 | 1.01(0.92~  1.11) | 0.781 | 1.08(1.05~  1.11) | <0.001 |
| Ethnic background |  |  |  |  |  |  |  |  |  |  |
| [European](file:///C:\Users\hzhy\Desktop\CHD\main%20document\javascript:;) | 1.08(1.06~  1.11) | <0.001 | 1.15(1.09~  1.22) | <0.001 | 1.15(1.11~  1.19) | <0.001 | 1.04(0.98~  1.11) | 0.163 | 1.10(1.08~  1.12) | <0.001 |
| Non-[European](file:///C:\Users\hzhy\Desktop\CHD\main%20document\javascript:;) | 1.09(1.00~  1.18) | 0.052 | 0.95(0.61~  1.36) | 0.788 | 1.14(0.98~  1.31) | 0.087 | 0.85(0.59~  1.14) | 0.313 | 1.06(0.98~  1.14) | 0.173 |

Abbreviation: HBP hypertension, HF heart failure, CAD coronary artery disease, CVD cardiovascular diseases, CM child maltreatment.

Table S9 Effect size (stratified by categorical covariates) of CM on two CVD comorbidities in full cohort (odd ratios and 95% CIs).

| Subgroup | HBP+HF  OR (95%CI) | *P* value | HBP+CAD  OR (95%CI) | *P* value | HBP+Stroke  OR (95%CI) | *P* value | HF+CAD  OR (95%CI) | *P* value | HF+Stroke  OR (95%CI) | *P* value | CAD+Stroke  OR (95%CI) | *P* value |
| --- | --- | --- | --- | --- | --- | --- | --- | --- | --- | --- | --- | --- |
| Maternal smoking around birth |  |  |  |  |  |  |  |  |  |  |  |  |
| No | 1.17(1.05~  1.29) | 0.003 | 1.11(0.98~  1.26) | 0.105 | 1.07(0.84~  1.34) | 0.586 | 1.19(0.95~  1.48) | 0.128 | 0.50(0.17~  1.16) | 0.146 | 0.83(0.50~  1.33) | 0.469 |
| Yes | 1.05(0.90~  1.22) | 0.485 | 1.06(0.85~  1.3) | 0.603 | 1.13(0.77~  1.63) | 0.515 | 1.16(0.80~  1.66) | 0.428 | 1.58(0.59~  3.93) | 0.339 | 1.1(0.45~  2.41) | 0.827 |
| Family history |  |  |  |  |  |  |  |  |  |  |  |  |
| No | 1.12(0.97~  1.27) | 0.107 | 1.04(0.87~  1.24) | 0.674 | 0.94(0.66~  1.31) | 0.712 | 1.24(0.91~  1.68) | 0.17 | 0.92(0.30~  2.34) | 0.875 | 0.85(0.43~  1.56) | 0.624 |
| Yes | 1.16(1.03~  1.28) | 0.008 | 1.14(1.00~  1.31) | 0.056 | 1.18(0.92~  1.5) | 0.182 | 1.15(0.90~  1.46) | 0.246 | 0.78(0.32~  1.68) | 0.558 | 0.92(0.51~  1.56) | 0.765 |
| Qualifications |  |  |  |  |  |  |  |  |  |  |  |  |
| University | 1.11(0.95~  1.28) | 0.181 | 1.14(0.95~  1.35) | 0.150 | 1.16(0.84~  1.58) | 0.344 | 1.35(1.01~  1.79) | 0.038 | 0.65(0.15~  1.95) | 0.491 | 1.05(0.54~  1.91) | 0.866 |
| Below university | 1.17(1.02~  1.31) | 0.016 | 1.05(0.89~  1.24) | 0.540 | 0.85(0.61~  1.16) | 0.316 | 1.19(0.89~  1.58) | 0.235 | 0.94(0.37~  2.06) | 0.878 | 0.66(0.32~  1.25) | 0.233 |
| Others | 1.14(0.94~  1.34) | 0.159 | 1.15(0.88~  1.49) | 0.295 | 1.68(1.06~  2.61) | 0.024 | 0.79(0.44~  1.32) | 0.381 | 0.87(0.13~  3.59) | 0.858 | 1.27(0.40~  3.43) | 0.658 |
| gender |  |  |  |  |  |  |  |  |  |  |  |  |
| Female | 1.17(1.03~  1.31) | 0.012 | 1.14(0.95~  1.36) | 0.163 | 1.32(0.99~  1.75) | 0.053 | 1.2(0.86~  1.65) | 0.279 | 1.03(0.40~  2.29) | 0.955 | 0.74(0.33~  1.45) | 0.406 |
| Male | 1.12(0.99~  1.26) | 0.057 | 1.08(0.94~  1.24) | 0.247 | 0.91(0.69~1.20) | 0.533 | 1.19(0.93~1.49) | 0.153 | 0.67(0.23~  1.60) | 0.411 | 0.99(0.58~1.61) | 0.960 |
| Ethnic background |  |  |  |  |  |  |  |  |  |  |  |  |
| [European](file:///C:\Users\hzhy\Desktop\CHD\main%20document\javascript:;) | 1.15(1.05~  1.25) | 0.001 | 1.11(0.99~  1.24) | 0.065 | 1.09(0.88~  1.32) | 0.424 | 1.17(0.97~  1.42) | 0.101 | 0.83(0.42~  1.52) | 0.576 | 0.92(0.59~  1.37) | 0.683 |
| Non-[European](file:///C:\Users\hzhy\Desktop\CHD\main%20document\javascript:;) | 0.89(0.46~  1.47) | 0.687 | 0.93(0.48~  1.72) | 0.824 | 1.43(0.36~  5.13) | 0.582 | 2.15(0.50~  9.32) | 0.289 | 1.00(0.98~  1.02) | 0.991 | 1.00(0.98~  1.02) | 0.996 |

Abbreviation: HBP hypertension, HF heart failure, CAD coronary artery disease, CVD cardiovascular diseases, CM child maltreatment.

Table S10 Effect size (stratified by categorical covariates) of CM on three or four CVD comorbidities in full cohort (odd ratios and 95% CIs).

| Subgroup | HBP+HF  +CAD  OR (95%CI) | *P*  value | HBP+HF  +Stroke  OR (95%CI) | *P*  value | HBP+CAD  +Stroke  OR (95%CI) | *P*  value | HF+CAD  +Stroke  OR (95%CI) | *P*  value | HBP+HF  +CAD+Stroke  OR (95%CI) | *P* value |
| --- | --- | --- | --- | --- | --- | --- | --- | --- | --- | --- |
| Maternal smoking around birth |  |  |  |  |  |  |  |  |  |  |
| No | 1.10(0.80~  1.49) | 0.553 | 0.68(0.20~  1.80) | 0.488 | 0.91(0.47~  1.62) | 0.759 | 0.25(0.04~  0.85) | 0.062 | 0.2(0.01~  0.97) | 0.118 |
| Yes | 1.24(0.78~  1.93) | 0.354 | 2.68(0.74~  9.66) | 0.120 | 1.13(0.36~  3.07) | 0.815 | 0.84(0.12~  3.49) | 0.828 | 0.73(0.04~  4.98) | 0.782 |
| Family history |  |  |  |  |  |  |  |  |  |  |
| No | 1.05(0.67~  1.59) | 0.835 | 1.25(0.34~  3.74) | 0.710 | 0.43(0.13~  1.1) | 0.117 | 0.27(0.01~  1.37) | 0.208 | 0.79(0.01~  1.57) | 0.990 |
| Yes | 1.22(0.88~  1.66) | 0.222 | 1.08(0.35~  2.77) | 0.875 | 1.41(0.74~  2.57) | 0.274 | 0.46(0.11~  1.32) | 0.205 | 0.84(0.07~  1.68) | 0.320 |
| Qualifications |  |  |  |  |  |  |  |  |  |  |
| University | 1.34(0.90~  1.96) | 0.135 | 0.43(0.02~  2.35) | 0.427 | 1.06(0.42~  2.35) | 0.893 | 0.54(0.08~  1.94) | 0.413 | 0.53(0.01~  1.49) | 0.993 |
| Below university | 1.12(0.75~  1.64) | 0.560 | 1.44(0.51~  3.6) | 0.452 | 0.83(0.36~  1.72) |  | 0.38(0.06~  1.31) | 0.190 | 0.63(0.1~  2.34) | 0.545 |
| Others | 0.83(0.39~  1.60) | 0.592 | 1.52(0.21~  7.8) | 0.630 | 1.14(0.25~  3.97) | 0.843 | 1.00(0.98~  1.02) | 0.992 | 1.28(0.01~  4.68) | 0.994 |
| gender |  |  |  |  |  |  |  |  |  |  |
| Female | 1.38(0.90~  2.07) | 0.127 | 1.51(0.53~  3.83) | 0.403 | 1.06(0.45~  2.26) | 0.878 | 0.24(0.01~  1.19) | 0.166 | 0.35(0.02~  1.81) | 0.312 |
| Male | 1.04(0.75~  1.43) | 0.799 | 0.79(0.18~  2.45) | 0.711 | 0.88(0.41~  1.7) | 0.719 | 0.5(0.12~  1.44) | 0.256 | 0.29(0.02~  1.46) | 0.230 |
| Ethnic background |  |  |  |  |  |  |  |  |  |  |
| [European](file:///C:\Users\hzhy\Desktop\CHD\main%20document\javascript:;) | 1.14(0.87~  1.47) | 0.333 | 1.15(0.51~  2.37) | 0.712 | 0.98(0.56~  1.63) | 0.948 | 0.39(0.12~  0.98) | 0.074 | 0.31(0.05~  1.06) | 0.116 |
| Non-[European](file:///C:\Users\hzhy\Desktop\CHD\main%20document\javascript:;) | 2.25(0.4~  12.56) | 0.331 | 1.00(0.98~  1.02) | 0.992 | 1.00(0.98~  1.02) | 0.998 | 1.00(0.98~  1.02) | 0.998 | 1.00(0.98~  1.02) | 0.998 |

Abbreviation: HBP hypertension, HF heart failure, CAD coronary artery disease, CVD cardiovascular diseases, CM child maltreatment.

**2.2 Supplementary Figures**


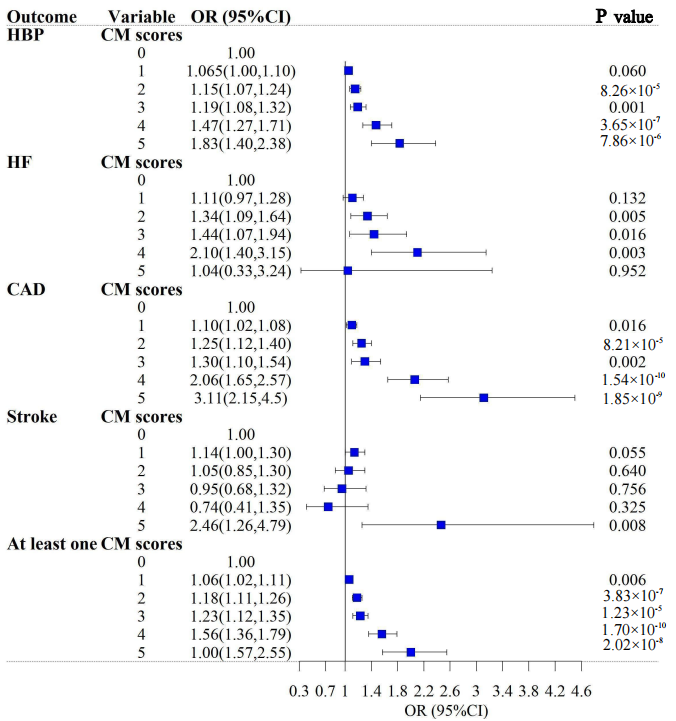


Figure S1 Dose-response relationships between CM scores and single or at least one CVD in all populations after adjustment for confounding variables (odd ratios and 95% CIs).

Abbreviation: HBP hypertension, HF heart failure, CAD coronary artery disease, CVD cardiovascular diseases, CM child maltreatment.


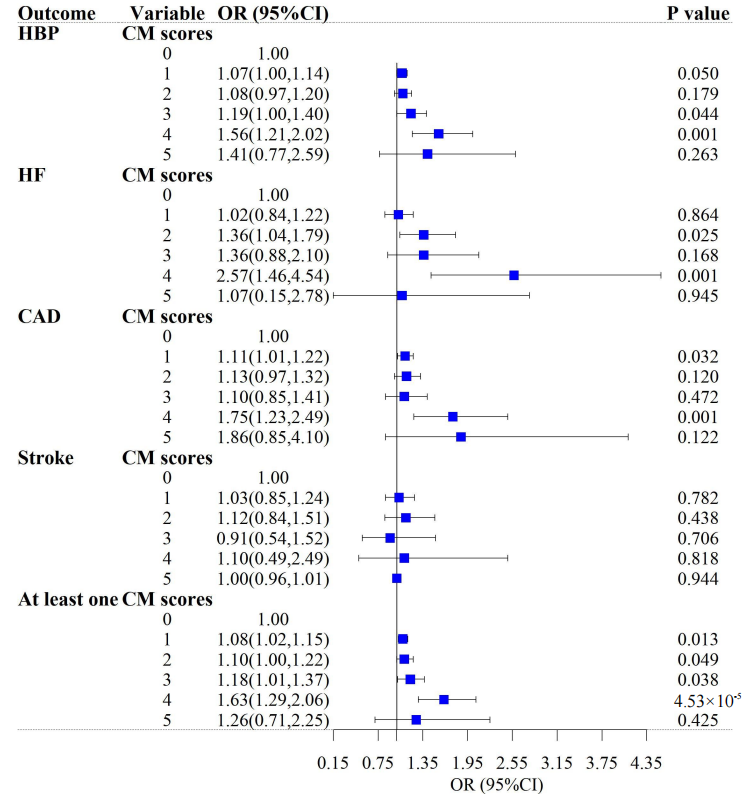


Figure S2 Dose-response relationships between CM scores and single or at least one CVD in males after adjustment for confounding variables (odd ratios and 95% CIs).

Abbreviation: HBP hypertension, HF heart failure, CAD coronary artery disease, CVD cardiovascular diseases, CM child maltreatment.


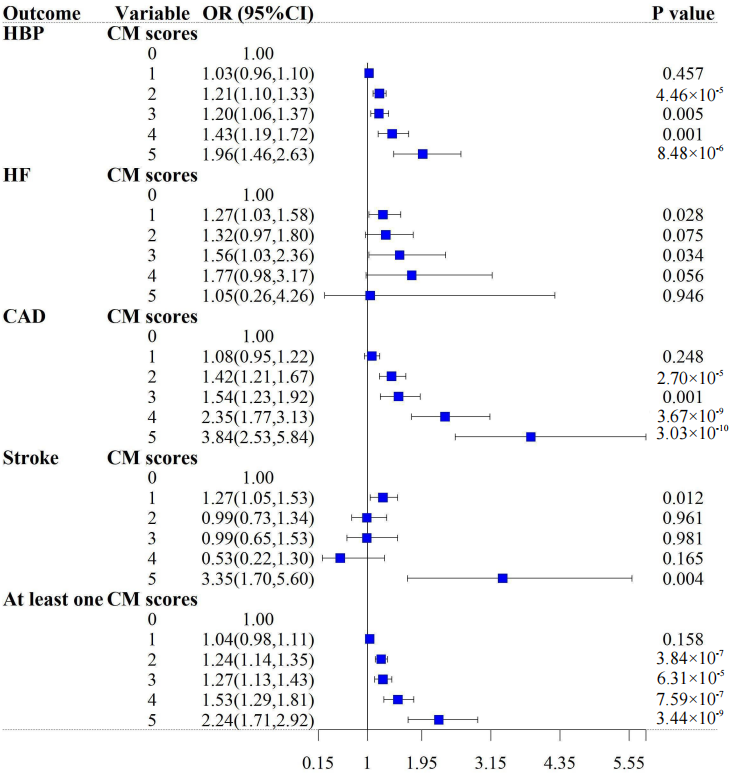


Figure S3 Dose-response relationships between CM scores and single or at least one CVD in females after adjustment for confounding variables (odd ratios and 95% CIs).

Abbreviation: HBP hypertension, HF heart failure, CAD coronary artery disease, CVD cardiovascular diseases, CM child maltreatment.


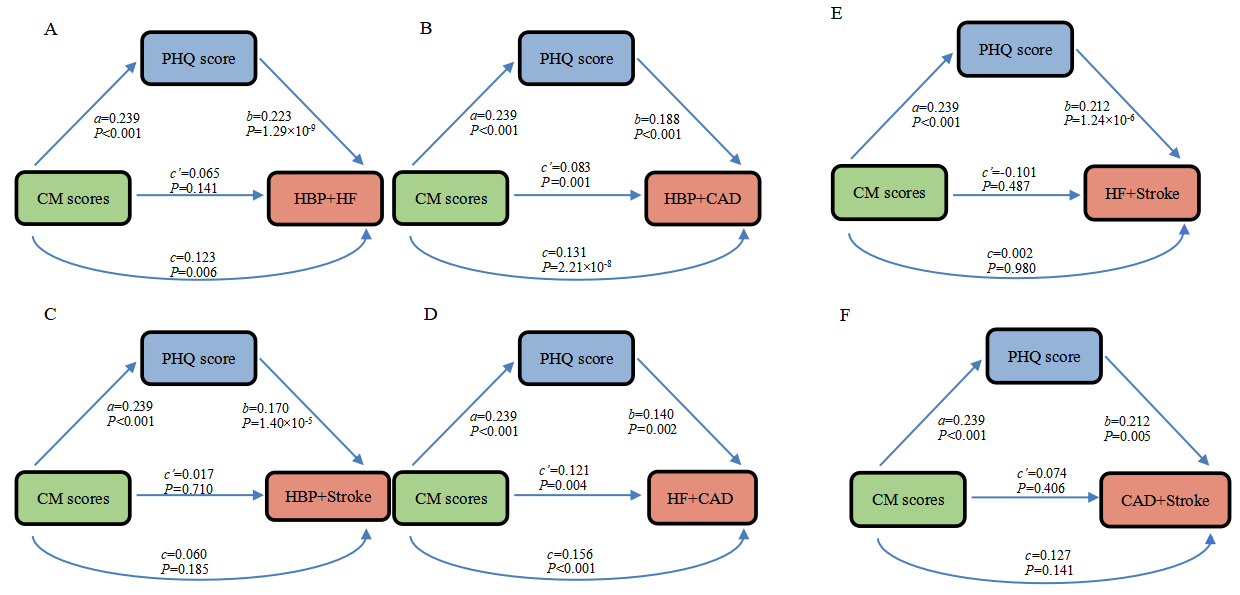


Figure S4 Mediation effect of depressive symptoms in the association between CM and two CVD comorbidities in all cohorts.

Abbreviation: HBP hypertension, HF heart failure, CAD coronary artery disease, CVD cardiovascular diseases, CM childhood maltreatment, PHQ-9 Patient Health Questionnaire 9-item.


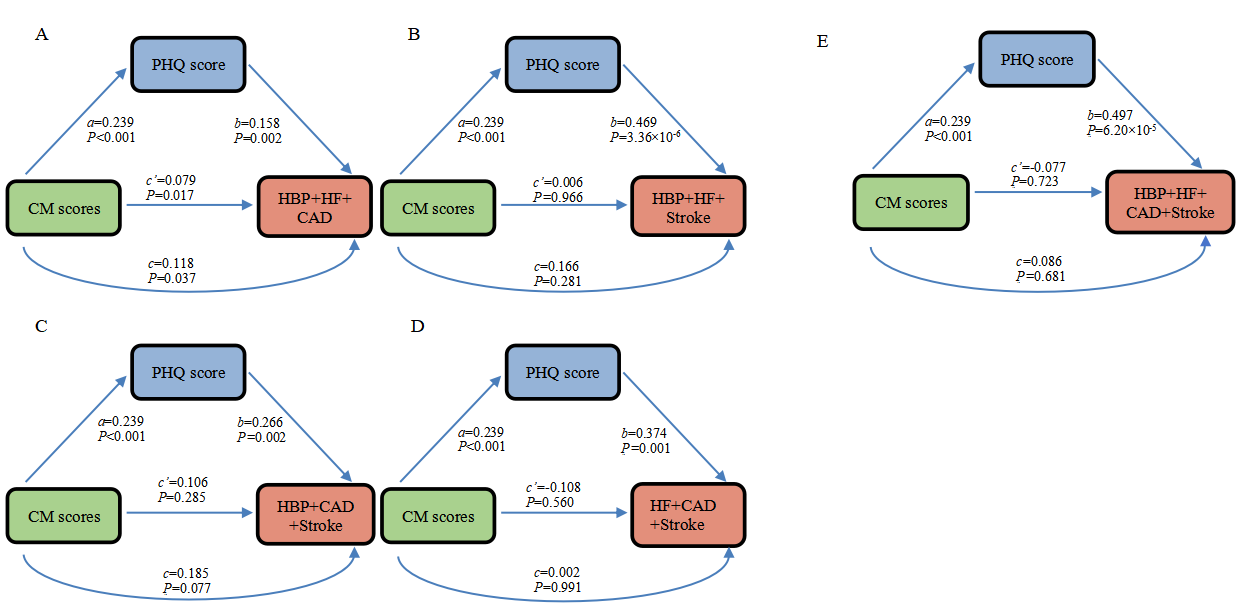


Figure S5 Mediation effect of depressive symptoms in the association between CM and three or four CVD comorbidities in all cohorts.

Abbreviation: HBP hypertension, HF heart failure, CAD coronary artery disease, CVD cardiovascular diseases, CM childhood maltreatment, PHQ-9 Patient Health Questionnaire 9-item.

CM scores

HBP

PHQ score

A

CM scores

HF

PHQ score

*a*=0.240

*P*<0.001

B

CM scores

CAD

PHQ score

*a*=0.240

*P*<0.001

*^4^*

*b*=0.211

*P*<0.001

C

CM scores

Stroke

PHQ score

*c’*=-1.42×10^-6^

*P*=0.991

*a*=0.240

*P*<0.001

*c*=0.044

*P*=0.276

D

*c’*=0.055

*P=*0.218

*b*=0.287

*P=*1.03×10^-15^

*c*=0.135

*P=*0.002

*b*=0.171

*P=*1.77×10^-6^

*c*=0.191

*P*<0.001

*c’*=0.136

*P=*4.28×10^-9^

*c*=0.084

*P=*9.40×10^-11^

*c’*=0.038

*P=*0.005

*b*=0.184

*P*<0.001

*a*=0.240

*P*<0.001

CM scores

At least one CVD

PHQ score

*a*=0.240

*P*<0.001

*^4^*

E

*b*=0.192

*P*<0.001

*c’*=0.154

*P*=1.08×10^-5^

*c*=0.103

*P<*0.001

Figure S6 Mediation effect of depressive symptoms in the association between CM and single or at least one CVD in females.

Abbreviation: HBP hypertension, HF heart failure, CAD coronary artery disease, CVD cardiovascular diseases, CM child maltreatment, PHQ-9 Patient Health Questionnaire 9-item.

CM scores

HBP

PHQ score

A

CM scores

HF

PHQ score

*a*=0.239

*P*<0.001

B

CM scores

CAD

PHQ score

*a*=0.239

*P*<0.001

*^4^*

*b*=0.123

*P*=4.02×10^-9^

C

CM scores

Stroke

PHQ score

*c’*=-0.020

*P*=0.685

*a*=0.239

*P*<0.001

*c*=0.009

*P*=0.850

D

*b*=0.120

*P=*0.003

*c*=0.065

*P*=8.51×10^-5^

*c*=0.135

*P=*0.002

*b*=0.082

*P*=0.054

*c’*=0.028

*P=*0.102

*c’*=0.116

*P=*0.008

*c*=0.081

*P=*0.001

*c’*=0.051

*P=*0.034

*b*=0.151

*P*<0.001

*a*=0.239

*P*<0.001

CM scores

At least one CVD

PHQ score

*a*=0.239

*P*<0.001

*^4^*

E

*b*=0.155

*P*<0.001

*c*’=0.031

*P*=0.039

*c*=0.070

*P*=3.40×10^-6^

Figure S7 Mediation effect of depressive symptoms in the association between CM and single or at least one CVD in males.

Abbreviation: HBP hypertension, HF heart failure, CAD coronary artery disease, CVD cardiovascular diseases, CM child maltreatment, PHQ-9 Patient Health Questionnaire 9-item.
